# Supplementary material for: Assessing inertial measurement unit locations for freezing of gait detection and patient preference
Source: J Neuroeng Rehabil. 2022 Feb 13;19:20. doi: 10.1186/s12984-022-00992-x (PMC8842967; doi:10.1186/s12984-022-00992-x)
Supplement: Supplementary file 1 — Additional file 1: Supplementary Information, figures S1 and S2. [file 12984_2022_992_MOESM1_ESM.docx]

**Additional file**


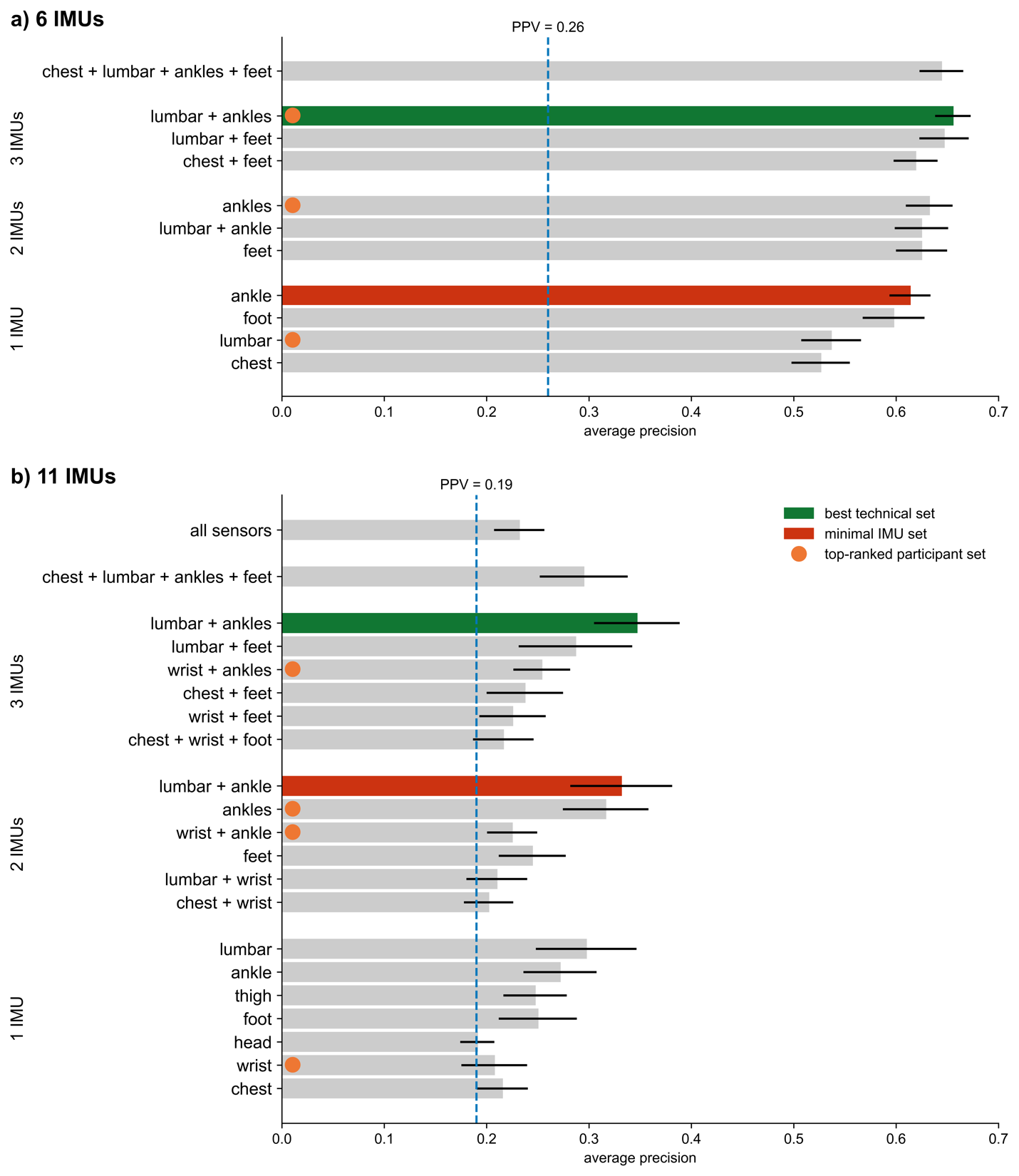


**Figure S1. Test set average precisions across subsets of the (a) 6-IMU and (b) 11-IMU sets.** Best technical sets are indicated by green bars, and minimal IMU sets are indicated by red bars, as determined by AUROC. Orange circles indicate the survey’s top-ranked sets for 1-, 2-, and 3-IMU subsets. The vertical blue dashed lines mark the positive predictive values (PPVs). Average precisions greater than this value demonstrate performance better than that of a random model. Note, top-ranked sets differ across (a) and (b) because the 6-IMU set does not include wrist IMUs.


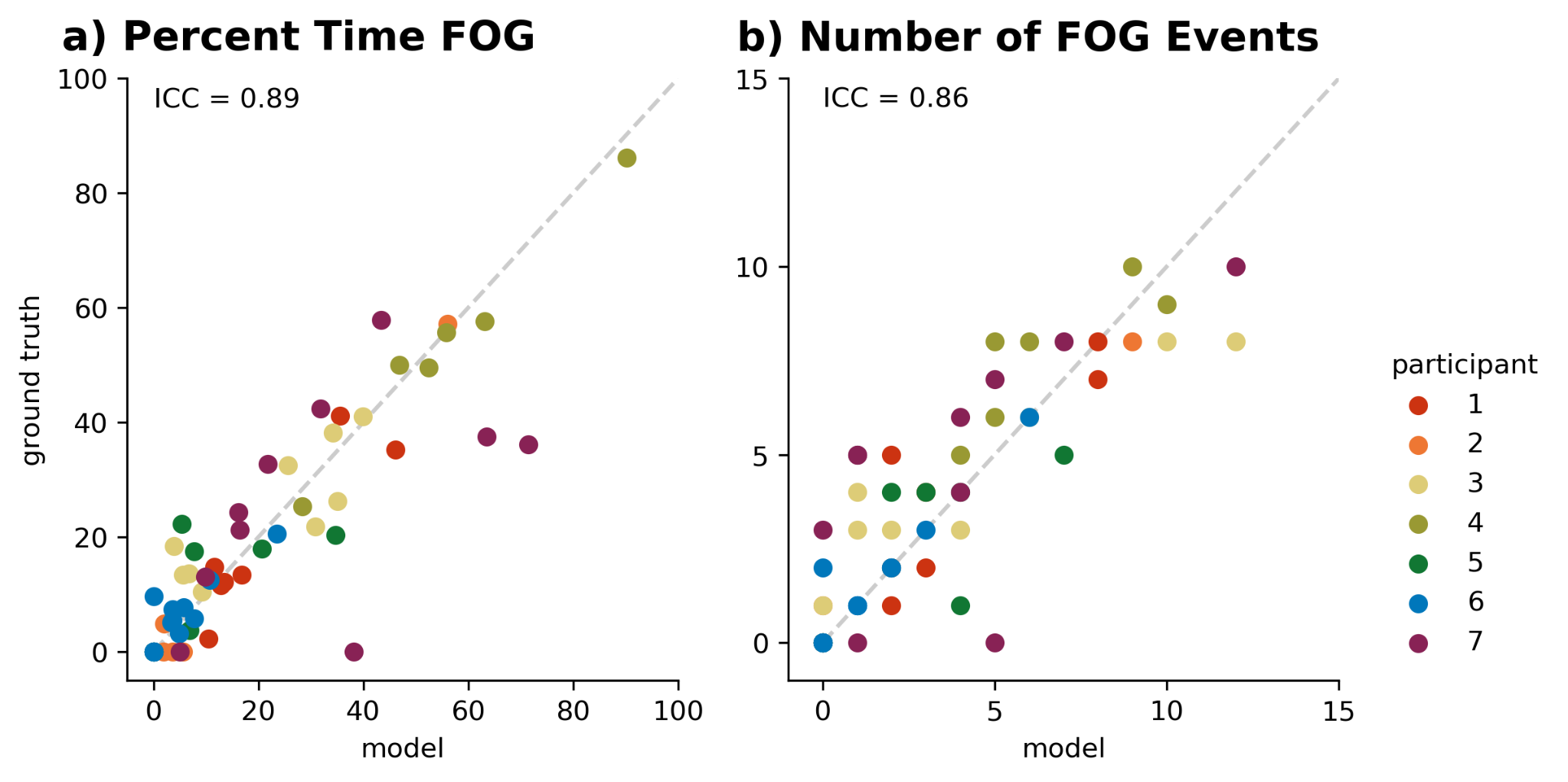


**Figure S2. Clinically-relevant metrics from a model using the minimal IMU set correlated with human ratings.** Human raters’ ground truth vs model predictions from the minimal sensor set (ankle) for (a) percent time FOG and (b) number of FOG events. Intraclass correlations (ICCs) for the two metrics were 0.89 and 0.86, respectively. Each walk is depicted by a single datapoint. Data from individual participants are depicted by color.
